# Polymorphism of the FaOMT and FaFAD1 genes for fruit flavor volatiles in strawberry varieties and wild species from the genetic collection of the Michurin Federal Research Center

**DOI:** 10.18699/VJ20.588

**Published:** 2020-02

**Authors:** A.S. Lyzhin, I.V. Luk’yanchuk, E.V. Zhbanova

**Affiliations:** I.V. Michurin Federal Scientific Center, Michurinsk, Tambov oblast, Russia; I.V. Michurin Federal Scientific Center, Michurinsk, Tambov oblast, Russia; I.V. Michurin Federal Scientific Center, Michurinsk, Tambov oblast, Russia

**Keywords:** strawberry, fruit aroma, mesifurane, γ-decalactone, molecular markers, FaOMT, FaFAD1, земляника, аромат плодов, мезифуран, γ-декалактон, молекулярные маркеры, гены FaOMT, FaFAD1

## Abstract

Fruit aroma is an important consumer attribute of strawberry varieties. The key volatile compounds of the aromatic complex of strawberry fruit are mesifurane (fruity and caramel aromas) and γ-decalactone (fruity, sweet, or peachy aroma). The mesifurane content in strawberry fruit is controlled by the FaOMT gene, which is mapped to the distal region of the long arm of chromosome VII-F.1. The γ-decalactone content in strawberry fruit is controlled by the FaFAD1 gene, mapped to the distal region of the long arm of chromosome III-2. Identification of forms carrying genes for fruit flavor volatiles is an important step in breeding varieties with fragrant fruit. The use of molecular markers allows highly reliable detection of target gene alleles in a genome at early developmental stages. This study involves molecular genotyping of Fragaria L. varieties for the FaOMT and FaFAD1 genes, analysis of polymorphism of the loci in question, and identification of genotypes valuable for breeding. The objects of our study were wild species of the genus Fragaria L. and strawberry varieties (Fragaria × ananassa Duch.) of different ecological and geographic origins. To assess the allelic states of the FaOMT gene, the codominant marker FaOMT-SI/NO was used, and for the FaFAD1 gene, the dominant marker FaFAD1. The functional allele of the FaOMT gene (FaOMT+) in the heterozygous state (FaOMT+FaOMT– genotype) was detected in 34.9 % of the accessions tested. The functional allele of the FaOMT gene in the homozygous state (FaOMT+FaOMT+ genotype) was detected in 51.2 % of the accessions. The homozygous state of the inactive allele (FaOMT–FaOMT– genotype) was detected in 13.9 % of the studied strawberry accessions. The FaFAD1 gene was identified in 25.6 % of the analyzed collection of strawberry genotypes, including the wild species F. orientalis Los., F. moschata Duch., F. ovalis Rydb. The combination of functional alleles of the FaOMT and FaFAD1 genes was detected in 16.3 % of the analyzed forms. The wild species F. orientalis Los. and F. moschata Duch. and strawberry variety Red Gauntlet combine the functional allele of the FaFAD1 gene with the homozygous state of the active allele of the FaOMT gene; therefore, we recommend them as promising sources of high contents of mesifurane and γ-decactone in fruit in breeding programs for fruit aroma.

## Abstract (ru)

Аромат плодов – важный потребительский признак сортов земляники. К числу ключевых компонентов ароматического комплекса плодов земляники относятся мезифуран (фруктовый и карамельный аромат) и γ-декалактон (персикоподобный, фруктовый, сладкий аромат). Содержание мезифурана в плодах земляники контролируется геном FaOMT, локализованным в дистальном районе длинного плеча хромосомы VII-F.1, γ-декалактона – геном FaFAD1, картированным в дистальном районе длинного плеча хромосомы III-2. Идентификация форм, несущих гены аромата, является важным этапом селекционных программ по созданию сортов с ароматными плодами. Использование молекулярных маркеров позволяет с высокой надежностью на ранних этапах онтогенеза определить присутствие в геноме целевых аллелей генов. Цель настоящего исследования – молекулярно-генетическое тестирование генотипов рода Fragaria L. по генам аромата плодов FaOMT и FaFAD1 для выявления полиморфизма изучаемых локусов и идентификации ценных для селекции генотипов. Объектами исследования были дикорастущие виды рода Fragaria L. и сорта земляники садовой (Fragaria × ananassa Duch.) различного эколого-географического происхождения. Для оценки аллельного состояния гена FaOMT использовали маркер FaOMT-SI/NO, гена FaFAD1 – маркер FaFAD1. Функциональный (активный) аллель гена FaOMT (FaOMT+) в гетерозиготном состоянии (генотип FaOMT+FaOMT–) выявлен у 34.9 % изучаемых форм, в гомозиготном (генотип FaOMT+FaOMT+) – у 51.2 %. Гомозиготное состояние неактивного аллеля (генотип FaOMT–FaOMT–) определено у 13.9 % образцов. Ген FaFAD1 в анализируемой коллекции генотипов земляники идентифицирован у 25.6 % форм, в том числе у дикорастущих видов F. orientalis Los., F. moschata Duch., F. ovalis Rydb. Сочетание функциональных аллелей генов FaOMT и FaFAD1 обнаружено у 16.3 % проанализированных форм. Дикорастущие виды F. orientalis Los., F. moschata Duch., а также сорт земляники садовой Red Gauntlet совмещают функциональный аллель гена FaFAD1 с гомозиготным состоянием активного аллеля гена FaOMT, что позволяет рекомендовать их в качестве перспективных комплексных источников высокого содержания мезифурана и γ-декалактона в плодах для селекции на аромат.

## Introduction

Strawberry (Fragaria × ananassa Duch.) is the most popular and economically important berry crop characterized by high taste and aroma of the fruits1 (Hummer, Hancock, 2009; Vandendriessche et al., 2013). Until recently, fruit aroma was not considered significant; therefore, many highly productive commercial varieties have feeble fruit aroma (Ulrich, Olbricht, 2016; Bianchi et al., 2017). Currently, due to the insistence on high standards, not only the taste but also the aroma of fruit, more attention is paid to creating varieties with improved fruit aroma (Ulrich, Olbricht, 2011; Zorrilla-Fontanesi et al., 2012).

Valuable source materials for strawberry breeding, including the breeding for fruit aroma, are wild species of the genus Fragaria L. Introgression of genes from wild strawberry species into the germ plasm of cultivated varieties F. × ananassa Duch. is expected to give rise to whole new genetic material and to expand the genetic polymorphism of breeding populations and the range of variation of traits, contributing to the acceleration of strawberry breeding (Hancock et al., 2010; Finn et al., 2013).

The aromatic profile of strawberry fruits is highly complex. It includes more than 350 volatile compounds: esters, furanones, terpenes, aldehydes, ketones, alcohols, sulfur compounds, etc. (Aharoni et al., 2004; Jetti et al., 2007; Schwab et al., 2008). The most important components of strawberry fruit aroma are furanones; in particular, furaneol (2,5-dimethyl-4-hydroxy-3(2H)-furanone) and its derivative mesifurane (2,5-dimethyl-4-methoxy-3(2H)-furanone). Furaneol and mesifurane contribute to fruit caramel aroma. The more furanones are contained in strawberry fruits, the sweeter is their aroma (Lavid et al., 2002; Raab et al., 2006). Another compound important for strawberry fruit aroma is γ-decalactone. This volatile contributes to fruity, sweet, or peachy aroma (Jouquand et al., 2008; Schwab et al., 2008). The concentrations of mesifurane and γ-decalactone in strawberry fruit are highly dependent on the genotype, environmental conditions, and the degree of fruit maturity (Ménager et al., 2004; Jetti et al., 2007; Olbricht et al., 2008; Siegmund et al., 2010). Moreover, unlike most components of the aromatic complex of strawberry fruits, whose biosynthesis is determined quantitatively, the contents of mesifuran and γ-decalactone are controlled by the dominant FaOMT and FaFAD1 genes, respectively. Therefore, functional DNA markers can be applied to effective screening of genotypes with high levels of the target traits, which allows highly reliable identification of carriers of target gene alleles at early developmental stages (Zorrilla-Fontanesi et al., 2012; Chambers et al., 2014; Sánchez-Sevilla et al., 2014).

The objectives of this study were the molecular genotyping of plants of the genus Fragaria L. for the FaOMT and FaFAD1 fruit flavor volatile genes, analysis of polymorphism for the loci of interest, and identification of valuable genotypes in breeding for fruit aroma.

## Materials and methods

Experiments were conducted with wild species and commercial varieties of strawberry from the genetic collection of the Michurin Federal Research Center, including 4 wild species of the genus Fragaria L., Kupchikha variety (F. × anashata Kantor.), and 38 strawberry varieties (Fragaria × ananassa Duch.), of which 22 genotypes were bred in Russia and 16 genotypes, outside Russia (Table 1).

**Table 1.**
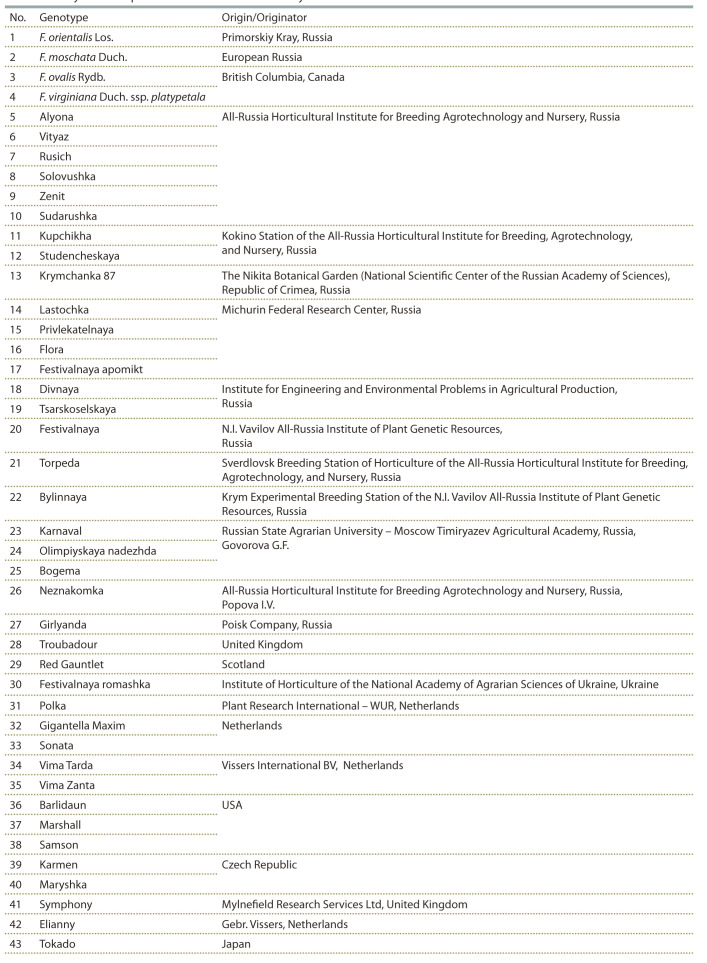
Analyzed wild species and varieties of strawberry

Total genomic DNA was extracted from fresh leaves using the Diversity Arrays Technology P/L (DArT, 2014) modified as in (Luk’yanchuk et al., 2018).

To assess the FaOMT allelic state, the codominant marker FaOMT-SI/NO (Zorrilla-Fontanesi et al., 2012) was used. The FaFAD1 gene was identified with the dominant marker FaFAD1 (Chambers et al., 2014). Primers used in this study were synthesized by Syntol (Russia). Sequences:

- FaOMT-SI/NO F 5′-CGATCATTTCGAAAAGGACTA- 3′, R 5′-AAGCAGGGTTAGTTGTGGAGA-3′;
- FaFAD1 F 5′-CGGGATTAATGGTTTTGTTGTTGACCGACC- 3′, R 5′-GTAGAAGAGAGAGACCAAGACGAG-3′.

PCR reactions were conducted in 15 μL of the amplification mixture containing 20 ng of genomic DNA, 0.2 mM of each dNTP, 2.5 mM MgCl2, 0.2 μM each primer, 0.2 U of Taq DNA polymerase, and 1.5 μL of PCR-buffer (+(NH4)2SO4, -KCl). All components were purchased from Thermo Fisher Scientific.

The amplification was performed in a T100 Thermal Cycler (BioRad). The PCR conditions for the FaOMT-SI/NO marker were as described by Cruz-Rus et al. (2017): predenaturation at 95 °C for 3 min followed by 10 cycles of 95 °C for 30 s, 60 °C (–0.5 °C/cycle) for 30 s, and 72 °C for 45 s; then 25 cycles of 95 °C for 30 s, 55 °C for 30 s, and 72 °C for 45 s; postextension at 72 °C for 5 min.

PCR conditions for the FaFAD1 marker were as described by Chambers et al. (2014): predenaturation at 94 °C for 4 min followed by 25 cycles of 94 °C for 30 s, 56 °C for 30 s, 72 °C for 30 s; postextension at 72 °C for 10 min.

The amplification products were resolved in 2 % agarose gel and visualized by ethidium bromide staining. GeneRuler 100 bp DNA Ladder (Thermo Fisher Scientific) was used as a molecular weight marker.

## Results and discussion

The mesifurane content in strawberry fruit is controlled by the FaOMT gene, which is mapped to the distal region of the long arm of the chromosome VII-F.1. The difference between the functional and nonfunctional alleles of the FaOMT gene is due to several single-nucleotide insertions/deletions (indels) in the promoter region of the gene, whose sizes total 30 bp. Primers FaOMT-SI/NO flanking the region with indels allow identification of the active (fragment of 248 bp) and inactive (fragment of 217 bp) FaOMT alleles (Zorrilla-Fontanesi et al., 2012). The effect of the FaOMT gene on mesifurane concentration was analyzed in a 232 × 1392 segregating population, where both parent forms were characterized by high mesifurane concentrations in the fruit. Statistical analysis of the results confirmed the participation of a single locus in the formation of the trait (expected 3:1 ratio, p = 0.36). We also analyzed the expression level of FaOMT in ripe fruits of forms with contrasting mesifurane contents. This analysis showed high FaOMT expression in forms with mesifurane-rich fruit and barely detectable expression in forms lacking mesifurane in fruit. This result supports the key role of the FaOMT gene in mesifurane content variation in strawberry fruit (Zorrilla- Fontanesi et al., 2012).

In the strawberry collection analyzed, the functional (active) allele of the FaOMT gene (FaOMT+) was identified in 86.1 % of forms out of 43 analyzed genotypes. The nonfunctional (inactive) allele (FaOMT–) was identified in 48.8 % forms out of 43 analyzed genotypes. The combination of active and inactive alleles of the FaOMT gene (FaOMT+FaOMT– genotype) was detected in 34.9 % of the analyzed forms. The homozygous state of the active allele of the FaOMT gene (FaOMT+FaOMT+ genotype) was identified in 51.2 % of the analyzed forms, and the homozygous state of the inactive allele (FaOMT–FaOMT–), in 13.9 %. An example of FaOMT allelic state analysis is shown in the Figure, a, and the overall results are summarized in Table 2.

**Fig. 1.**
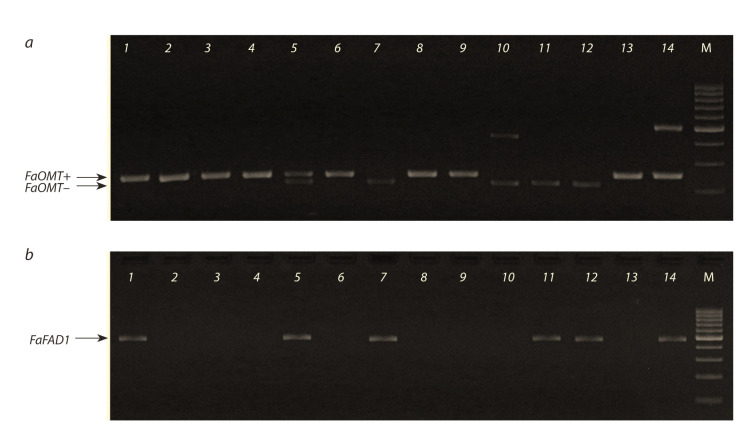
Electrophoresis profiles of markers (a) FaOMT-SI/NO and (b) FaFAD1 at strawberry genotypes. Lanes: 1, Red Gauntlet; 2, Lastochka; 3, Torpeda; 4, Zenit; 5, Sonata; 6, Karmen; 7, Bylinnaya; 8, Samson; 9, Bogema; 10, Sudarushka; 11, Kupchikha; 12, F. ovalis Rydb.; 13, F. virginiana Duch. ssp. platypetala; 14, F. moschata Duch.; М, molecular weight ladder.

**Table 2.**
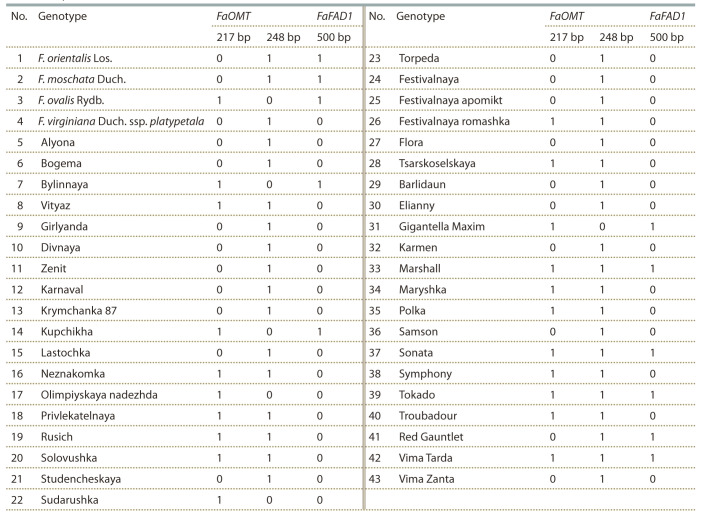
Allelic diversity of the FaOMT and FaFAD1 fruit flavor volatile genes in strawberry varieties and wild species (1, allele is present; 0, absent)

Among the analyzed wild species of the genus Fragaria L., the FaOMT+ allele (FaOMT+FaOMT+ genotype) was detected in F. orientalis Los., F. moschata Duch., and F. virginiana Duch. ssp. platypetala. It should be noted that the target products of the FaOMT-SI/NO marker are not amplified in French variety Capron Royale (F. moschata Duch.) with high fruit mesifurane content (Cruz-Rus et al., 2017). This result might be due to substitutions at the primer-binding site or the effect of other genetic factors on mesifurane content. It requires additional studies.

Among the 22 Russian strawberry varieties analyzed, the homozygous state of the functional FaOMT+ allele was identified in 59.1 % of forms and the heterozygous combination, in 27.3 %. The homozygous state of the nonfunctional FaOMT– allele was identified in 13.6 % of Russian straw-berry varieties. Among the analyzed 16 foreign strawberry varieties, 56.3 % of forms had the FaOMT+FaOMT– genotype, 37.5 % forms had the FaOMT+FaOMT+ genotype, and 6.2 % forms had the FaOMT–FaOMT– genotype. The predominance of the heterozygous combination alleles of the FaOMT gene in foreign strawberry varieties is consistent with literature data (Cruz-Rus et al., 2017).

The γ-decalactone content in strawberry fruit is controlled by the FaFAD1 gene (candidate gene 24414), which is mapped to the distal region of the long arm of chromosome III-2 in the F. × ananassa Duch. genome (Sánchez-Sevilla et al., 2014). Comparison of the genomes of the Elyana variety (γ-decalactone is produced) and the Mara des Bois variety (γ-decalactone is not produced) shows that the γ-decalactone content in strawberry fruit is determined by the expression of one functional FaFAD1 allele, and the absence of γ-decalactone in fruit is caused either by mRNA FaFAD1 gene transcription block, or by the lack of the active allele from the genome (Chambers et al., 2014).

Primers FaFAD1-F/R amplify a 500 bp fragment at the 5′ end of gene 24414. This fragment is not amplified in genotypes with undetectable γ-decalactone in fruit (Chambers et al., 2014). The relationship between the presence of the functional FaFAD1 allele in the genome and the γ-decalactone content in fruit was tested on three hybrid combinations: Elyana (γ-decalactone is produced) × Mara des Bois (γ-decalactone is not produced), Elyana (γ-decalactone is produced) × 98 (γ-decalactone is produced), and Mara des Bois (γ-decalactone is not produced) × 98 (γ-decalactone is produced). All strawberry genotypes with high γ-decalactone contents in the fruit possessed the functional FaFAD1 allele. The correlation between the presence of the functional FaFAD1 allele and γ-decalactone presence in the fruit was also confirmed by analysis of γ-decalactone-producing varieties Radiance, Albion, Winter Star, and Sweet Charlie and non-γ-decalactone-producing varieties Deutsch Evern, Strawberry Festival, LF9, and Mieze Schindler (Chambers et al., 2014). As reported in (Zorrilla-Fontanesi et al., 2012), in 93.3 % of cases high γ-decalactone content in fruit is due to the presence of the FaFAD1 gene.

In the analyzed collection of 43 strawberry genotypes, the FaFAD1 gene was identified in 25.6 % forms, including the wild species F. orientalis Los., F. moschata Duch., and F. ovalis Rydb. An example of FaFAD1 gene identification is shown in the Figure, b, and the results are shown in Table 2. The FaFAD1 gene is also present in the French variety Capron Royale (F. moschata Duch.) (Cruz-Rus et al., 2017). Among the 22 analyzed Russian strawberry varieties, the FaFAD1 gene was identified in 9.1 % of forms (varieties Bylinnaya and Kupchikha). Among the analyzed 16 foreign strawberry varieties, FaFAD1 was identified in 37.5 % forms. According to the data of Cruz-Rus et al. (2017), the FaFAD1 gene was identified in 40.0 % of tested strawberry genotypes (F. × ananassa Duch.) of non-Russian breeding.

The wider distribution of the FaFAD1 gene in the germ plasm of non-Russian strawberry varieties is presumably explained by the genetic proximity of many varieties due to the widespread use of the same parental forms in breeding (Most of the non-Russian strawberry varieties created after 1960 were obtained by crosses of seven parental forms (Lei et al., 2002).), and one or more of these forms could be a donor of the functional allele of the FaFAD1 gene.

Strawberry fruit aroma is a complex multicomponent trait, whose manifestation is determined by the expression of many genetic factors. In this regard, the most promising forms in breeding for fruit aroma are genotypes with several fruit flavor volatile genes in the genome. In the analyzed collection of strawberry genotypes, the combination of functional alleles of the FaOMT and FaFAD1 genes was detected in 16.3 % of the forms (see Table 2). Among them, the wild species F. orientalis Los., F. moschata Duch. and strawberry variety Red Gauntlet combine the functional allele of the FaFAD1 gene with the homozygous state of the active allele of the FaOMT gene. Foreign strawberry varieties Marshall, Sonata, Tokado, and Vima Tarda combined the functional allele of the FaFAD1 gene with the heterozygous state of the FaOMT gene. The combination the functional alleles of the FaFAD1 and FaOMT genes was not found in the analyzed Russian strawberry varieties.

## Conclusion

Thus, according to the results of molecular analysis of the FaOMT allelic state, the promising sources of high mesifurane content in breeding for fruit aroma are Russian strawberry varieties Alyona, Bogema, Girlyanda, Divnaya, Zenit, Karnaval, Krymchanka 87, Lastochka, Studencheskaya, Torpeda, Festivalnaya, and Flora, and foreign strawberry varieties Barlidaun, Elianny, Karmen, Samson, and Vima Zanta, which are characterized by the homozygous state of the functional allele of the FaOMT gene (FaOMT+FaOMT+ genotype). The sources of high γ-decalactone content in fruit are varieties Bylinnaya, Kupchikha, Gigantella Maxim, Marshall, Sonata, Tokado, and Vima Tarda, which are characterized by the presence of the active allele of the FaFAD1 gene. The wild species F. orientalis Los., F. moschata Duch., and strawberry variety Red Gauntlet, combining the functional allele of the FaFAD1 gene with the homozygous state of the active allele of the FaOMT gene, are complex sources of high mesifuran and γ-decalactone contents in fruit.

## Conflict of interest

The authors declare no conflict of interest.
